# Visceral Adiposity Associates With Malnutrition Risk Determined by Royal Free Hospital-Nutritional Prioritizing Tool in Cirrhosis

**DOI:** 10.3389/fnut.2021.766350

**Published:** 2021-11-24

**Authors:** Xiaoyu Wang, Yifan Li, Mingyu Sun, Gaoyue Guo, Wanting Yang, Yangyang Hui, Zihan Yu, Chaoqun Li, Xiaofei Fan, Bangmao Wang, Jie Zhang, Xingliang Zhao, Kui Jiang, Chao Sun

**Affiliations:** ^1^Department of Gastroenterology and Hepatology, Tianjin Medical University General Hospital, Tianjin, China; ^2^Tianjin Institute of Digestive Disease, Tianjin Medical University General Hospital, Tianjin, China; ^3^Department of Internal Medicine, Tianjin Hexi Hospital, Tianjin, China; ^4^Department of Gastroenterology, Tianjin Medical University General Hospital Airport Hospital, Tianjin, China

**Keywords:** visceral adiposity, cirrhosis, malnutrition, RFH-NPT, visceral to subcutaneous adipose tissue area ratio

## Abstract

Mounting evidence has suggested the clinical significance of body composition abnormalities in the context of cirrhosis. Herein, we aimed to investigate the association between visceral adiposity and malnutrition risk in 176 hospitalized patients with cirrhosis. The adiposity parameters were obtained by computed tomography (CT) as follows: total adipose tissue index (TATI), visceral adipose tissue index (VATI), subcutaneous adipose tissue index (SATI), and visceral to subcutaneous adipose tissue area ratio (VSR). Malnutrition risk was screened using Royal Free Hospital-Nutritional Prioritizing Tool (RFH-NPT). Visceral adiposity was determined given a higher VSR based on our previously established cutoffs. Multivariate analysis implicated that male gender (OR = 2.884, 95% CI: 1.360–6.115, *p* = 0.006), BMI (OR = 0.879, 95% CI: 0.812–0.951, P = 0.001), albumin (OR = 0.934, 95% CI: 0.882–0.989, *P* = 0.019), and visceral adiposity (OR = 3.413, 95% CI: 1.344–8.670, *P* = 0.010) were independent risk factors of malnutrition risk. No significant difference was observed regarding TATI, SATI, and VATI among patients with low or moderate and high risk of malnutrition. In contrast, the proportion of male patients embracing visceral adiposity was higher in high malnutrition risk group compared with that in low or moderate group (47.27 vs. 17.86%, *p* = 0.009). Moreover, this disparity was of borderline statistical significance in women (19.05 vs. 5.88%, *p* = 0.061). Assessing adipose tissue distribution might potentiate the estimation of malnutrition risk in cirrhotics. It is pivotal to recognize visceral adiposity and develop targeted therapeutic strategies.

## Introduction

Malnutrition is prevalent in patients with cirrhosis, which contributes to the increased risk of morbidity and mortality (1). It is of utmost importance to identify malnourished subjects and institute nutritional therapy with the purpose of reducing mortality, systemic inflammatory response, and infection (2, 3). The Royal Free Hospital-Nutritional Prioritizing Tool (RFH-NPT) is a cirrhosis-specific nutrition screening tool. In an established and validated cohort of 148 patients with chronic liver disease, the RFH-NPT represented a useful predictor of clinical deterioration and poor outcome (4). Our previous work also implicated that malnutrition risk estimated by RFH-NPT is dramatically associated with distorting immune function in the context of cirrhosis (5).

Evaluation of body composition, including muscle and adipose tissue, gives rise to an objective assessment of the patients' metabolic and nutritional status. Muscles are responsible for mechanical activity, whereas adipose tissue is involved in energy regulation and metabolic action (6). We and others have substantially clarified the prognostic utility of several abnormalities in body composition features for outcomes in patients with cirrhosis (7–9). More recently, Borges et al. found that sarcopenia (low muscle mass) serves as a predictor of malnourished condition and comorbidities in hospitalized patients with cancer (10). Furthermore, it has been documented that malnutrition determined by Patient-Generated Subjective Global Assessment (PG-SGA) is an indicator of sarcopenia in cirrhotics (11). However, the association between abnormal adiposity and malnutrition risk remains elusive in hospitalized patients with cirrhosis. The excessive depot of visceral adipose tissue might promote inflammation and metabolic dysregulation (12, 13). Likewise, failure to expand subcutaneous adipose tissue contributes to visceral fat deposition as well as insulin resistance (14, 15). Intriguingly, some investigations indicated that the distribution of adipose tissue rather than the absolute volume appears to be a major determinant for prognostication in various liver diseases (7, 16). Therefore, we aimed to investigate the association between visceral adiposity and malnutrition risk in hospitalized patients with cirrhosis.

## Methods

### Patients

**Figure 1:**
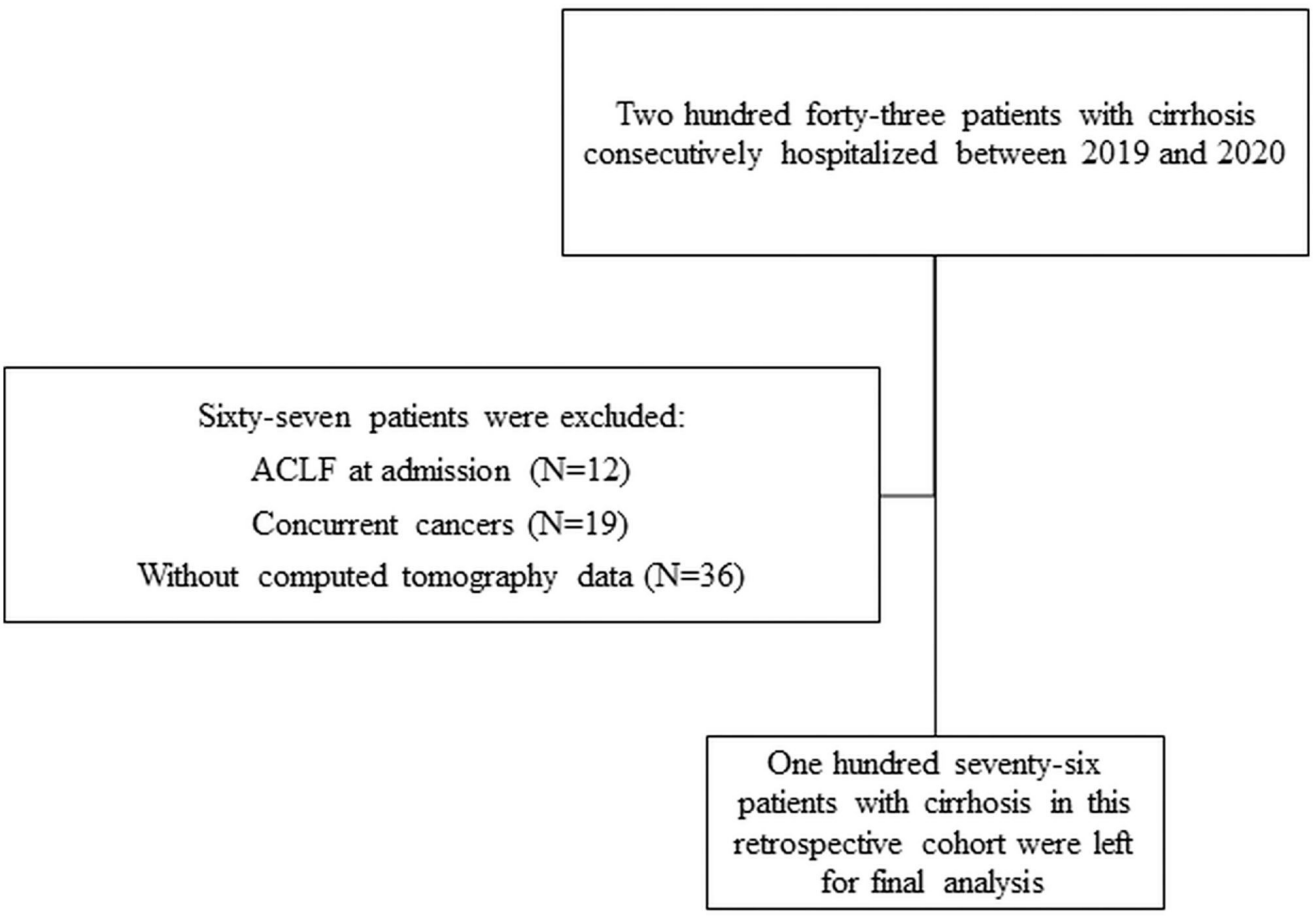
A flowchart of study population, acute on chronic liver failure (ACLF).

Among 243 adult patients aged not less than 18 years who were consecutively enrolled in Department of Gastroenterology and Hepatology, Tianjin Medical University General Hospital (TJMUGH) between 2019 and 2020, 12 with acute-on-chronic liver failure upon admission, 19 with concurrent cancers and 36 without CT scan during hospitalization were excluded from this work (Figure 1). Therefore, this retrospective cohort study explored and analyzed data from 176 patients {men, *n* = 83; women, *n* = 93; median age, 63 years [interquartile range (IQR), 56–68]}. Details of the diagnosis of liver cirrhosis, retrieval of laboratory results, and cirrhosis-associated complications have been comprehensively described elsewhere (17, 18). This work was conducted adherent to the Declaration of Helsinki, was approved by Ethics Committee of TJMUGH (2018-235), and was presented in accordance with the STROBE statement. Written informed consent was obtained from all participants.

### Assessment of Computed Tomography Images

All CT images of the study cohort were achieved using a spectral CT scanner (Discovery 750 HD 64 row, General Electric Company, Boston, USA). Details in relation to image analyses of distinct adipose tissues have been explicitly depicted in our previous publication (7). In brief, body composition was quantified using an opensource software based on the MATLAB version R2010a (Mathworks Inc., Natick, Massachusetts, USA). The tissue-specific attenuation values from−190 to−30 Hounsfield unit (HU) were for subcutaneous or visceral adipose tissue. Acquired values were standardized for height in squared meters (cm^2^/m^2^). Accordingly, several parameters were obtained as follows: total adipose tissue index (TATI), visceral adipose tissue index (VATI), and subcutaneous adipose tissue index (SATI). Visceral adiposity was evaluated by visceral to subcutaneous adipose tissue area ratio (VSR) (9, 16).

### Cutoff Values for VSR and Other Adiposity Parameters

The gender-specific cutoff values for body composition indices were generated separately in terms of our previous work by X-tile as follows: VSR (men, 1.47; women, 1.29), SATI (men, 29.10 cm^2^/m^2^; women, 26.75 cm^2^/m^2^), and VATI (men, 28.42 cm^2^/m^2^; women, 44.02 cm^2^/m^2^) (7). The X-tile project (Yale University School of Medicine, New Haven, Connecticut, USA) can attain a single, global estimation of each probable modality of dividing a cohort into low-level and high-level marker expressions.

### Royal Free Hospital-Nutritional Prioritizing Tool

We demonstrated the RFH-NPT score in Supplementary Figure S1. Generally speaking, it takes approximate 3 min to complete this scale, which includes the components of alcoholic hepatitis, fluid overload and influence on dietary intake, body mass index (BMI), and unplanned weight loss. Taken together, the RFH-NPT discriminates patients with cirrhosis into low- (0 points), medium- (1 point), and high-risk (2–7 points) categories.

### Statistical Analysis

Data were presented as mean ± standard deviation (SD), median (IQR), simple frequencies, or percentages (%) as appropriate. Continuous data were compared using an independent Student's *t*-test or the Mann–Whitney *U* test appropriately. Categorical variables were compared by χ^2^ test or Fisher's exact test. Multivariate analysis performed by logistic regression analysis was used to figure out the independent risk factor of high risk of malnutrition. Odds ratio (OR) and 95% confidence interval (CI) were calculated. All *p*-values were two-sided, and we regarded *p* < 0.05 as statistical significance. The statistical analyses were carried out using MedCalc 15.2.2 (MedCalc, Mariakerke, Belgium) and Stata 14.0 (Stata Corporation, College Station, Texas, USA).

## Results

Table 1 shows the baseline features and laboratory data of the 176 patients. The etiology of cirrhosis was attributed to chronic hepatitis B virus (HBV) or hepatitis C virus (HCV) infection in 45 (25.57%), alcohol in 45 (25.57%), autoimmune liver disease in 41 (23.29%), and nonalcoholic fatty liver disease (NAFLD) and cryptogenic reasons in 45 (25.57%) subjects, respectively. The cirrhosis-associated complications consisted of ascites in 105 (59.66%), esophagogastric varices in 123 (69.89%), infection in 27 (15.34%), and hepatic encephalopathy in 16 (9.09%), respectively. Among the study population, 43 (24.43%) were categorized into CTP class A, 104 (59.09%) into CTP class B, and 29 (16.48%) into CTP class C. The median MELD-Na score upon hospitalization was 10 (IQR, 6–13.75).

**Table 1:** Baseline characteristics of cirrhotic patients stratified according to RFH-NPT risk classification.

	**Total (*N* = 176)**	**RFH-NPT risk classification**		***P***
		**Low and Moderate (*N* = 79)**	**High (*N* = 97)**	
Age (years)	63 (56–68)	62 (53.75–67)	64 (57–69)	0.106
Gender, *n* (%)				0.005
Male	83 (47.16)	28 (35.44)	55 (56.70)	
Female	93 (52.84)	51 (64.56)	42 (43.30)	
CTP, *n* (%)				0.009
A	43 (24.43)	27 (34.18)	16 (16.49)	
B	104 (59.09)	44 (55.70)	60 (61.86)	
C	29 (16.48)	8 (10.12)	21 (21.65)	
MELD-Na score	10 (6–13.75)	9(7–12)	10 (6-15)	0.394
Etiology, *n* (%)				< 0.001
HBV/HCV	45 (25.57)	28 (35.44)	17 (17.53)	
Alcohol	45 (25.57)	4 (5.06)	41 (42.27)	
AILD	41 (23.29)	23 (29.11)	18 (18.56)	
NAFLD/Cryptogenic	45 (25.57)	24 (30.39)	21(21.64)	
Complications, *n* (%)			
Gastroesophageal varices	123 (69.89)	56 (70.89)	67 (69.07)	0.869
Hepatic encephalopathy	16 (9.09)	6 (7.59)	10 (10.31)	0.606
Ascites	105 (59.66)	33 (41.77)	72 (74.23)	< 0.001
Infection	27 (15.34)	9 (11.39)	18 (18.56)	0.213
BMI (kg/m^2^)	22.89 ± 4.72	24.28 ± 4.89	21.68 ± 4.24	< 0.001
Waist circumference (cm)	93.79 ± 14.38	91.75 ± 12.37	95.52 ± 15.73	0.094
VSR	0.99 (0.3–1.42)	0.92 (0.67–1.08)	1.17 (0.86–1.66)	< 0.001
Visceral adiposity	42 (23.86)	8 (10.13)	34 (35.05)	< 0.001
High visceral adiposity	118 (67.05)	50 (63.29)	68 (70.10)	0.420
Low subcutaneous adiposity	40 (22.73)	13 (16.46)	27 (27.84)	0.103
Platelet (×10^9^/L)	80 (55.25-114.80)	76 (47–113)	86 (60.50–119)	0.172
Albumin (g/L)	28 (24–32)	30 (25–34)	27 (23–30.50)	0.006
TBIL (μmol/L)	22.75 (14.40–38.90)	22.40 (13.60–36.14)	22.8 (14.9–44.7)	0.517
ALT (U/L)	23.50 (15–37)	25 (17–41)	23 (14–35.50)	0.072
AST (U/L)	31 (21.25–52.75)	31 (22-57)	31 (20.50–47.50)	0.657
Creatinine (μmol/L)	59 (49.25–73)	58 (47–66)	61 (51–82.50)	0.021
PT-INR	1.27 (1.17–1.46)	1.24 (1.17, 1.35)	1.31 (1.18–1.54)	0.023

*Values are presented as the mean ± standard deviation, median (IQR), or number of patients (%)*.

*RFH-NPT, Royal Free Hospital-Nutritional Prioritizing Tool; AILD, auto-immune liver disease; NAFLD, non-alcoholic fatty liver disease; CTP, Child-Turcotte-Pugh class; BMI, body mass index; VSR, visceral to subcutaneous ratio of adipose tissue area; MELD-Na, model for end-stage liver disease-sodium; TBIL, total bilirubin; PT-INR, prothrombin-international normalized ratio*.

Then, these patients were divided into two groups in terms of malnutrition risk determined by RFH-NPT score. Among them, 79 (44.89%) patients were in the low- or moderate-risk group and 97 (55.11%) were in the high-risk group. There were significant differences in gender, CTP class, etiology, the presence of ascites, BMI, the presence of sarcopenia, visceral adiposity, albumin, creatinine, and PT-INR. Patients with high risk of malnutrition were dominant in men, more CTP class B/C, more alcoholism, more ascites, lower BMI, more sarcopenia, more visceral adiposity, lower albumin, higher creatinine, and PT-INR.

### Independent Risk Factor of High Risk of Malnutrition

The univariate and multivariate analyses of malnutrition risk are shown in Table 2. Univariate analysis revealed that age (*p* = 0.041), male gender (*p* = 0.005), alcoholism (*p* < 0.001), CTP class (*p* = 0.011), ascites (*p* < 0.001), BMI (*p* = 0.001), sarcopenia (*p* = 0.007), visceral adiposity (*p* < 0.001), and albumin (*p* = 0.008) were significantly associated with high risk of malnutrition. Taking into consideration that alcoholic liver disease that exhibits a large weight on original RFH-NPT score, we decided to construct two multivariate logistic regression model. In model 1, our results indicated that visceral adiposity exhibits borderline significance (OR = 2.705, 95% CI: 0.968–7.557, *p* = 0.058). In model 2 excluding etiology, we found that male gender (OR = 2.884, 95% CI: 1.360–6.115, *p* = 0.006), BMI (OR = 0.879, 95% CI: 0.812–0.951, *p* = 0.001), albumin (OR = 0.934, 95% CI: 0.882–0.989, *p* = 0.019), and visceral adiposity (OR = 3.413, 95% CI: 1.344–8.670, *p* = 0.010) were independent risk factors of malnutrition risk determined by RFH-NPT in hospitalized patients with cirrhosis.

**Table 2:** Univariate and multivariate analysis for malnutrition risk determined by RFH-NPT.

**Variable**	**Univariate analysis**			**Multivariate analysis**					
				Model 1^*^			Model 2^*^		
	**OR**	**95% CI**	***P***	**OR**	**95% CI**	***P***	**OR**	**95% CI**	***P***
Age (years)	1.031	1.001, 1.062	0.041				1.033	0.999, 1.069	0.058
**Gender**							2.884	1.360, 6.115	**0.006**
Male	2.385	1.294, 4.396	0.005						
Female	Reference								
**Etiology**			< 0.001	0.650		**<0.001**			
HBV/HCV	0.694	0.299, 1.608	0.394	0.650	0.255, 1.658	0.367			
Alcohol	11.714	3.592, 38.198	< 0.001	9.994	2.846, 35.091	**<0.001**			
AILD	0.894	0.382, 2.094	0.797	0.804	0.306, 2.113	0.659			
NAFLD/Cryptogenic	Reference			Reference			Reference		
**CTP**			0.011						
A	Reference								
B	2.301	1.108, 4.778	0.025						
C	4.430	1.593, 12.315	0.004						
Ascites	4.015	2.121, 7.598	< 0.001						
BMI	0.890	0.830, 0.593	0.001	0.866	0.796, 0.942	**0.001**	0.879	0.812, 0.951	**0.001**
Visceral adiposity	4.790	2.065, 11.112	< 0.001	2.705	0.968, 7.557	0.058	3.413	1.344, 8.670	**0.010**
Albumin	0.933	0.887, 0.982	0.008	0.940	0.887, 0.996	**0.036**	0.934	0.882, 0.989	**0.019**

*Multivariate model 1: Age, gender, etiology, CTP class, BMI, ascites, visceral adiposity and albumin*;

* *Final model presented*.

*Multivariate model 2: Age, gender, CTP class, BMI, ascites, visceral adiposity and albumin*;

* *Final model presented*.

*RFH-NPT, Royal Free Hospital-Nutritional Prioritizing Tool; OR, odds ratio; CI, confidence interval; AILD, auto-immune liver disease; NAFLD, non-alcoholic fatty liver disease; CTP, Child-Turcotte-Pugh class; BMI, body mass index*.

*The bold values indicate statistical significance*.

### Gender-Stratified Analysis of Adipose Tissue and RFH-NPT

**Figure 2:**
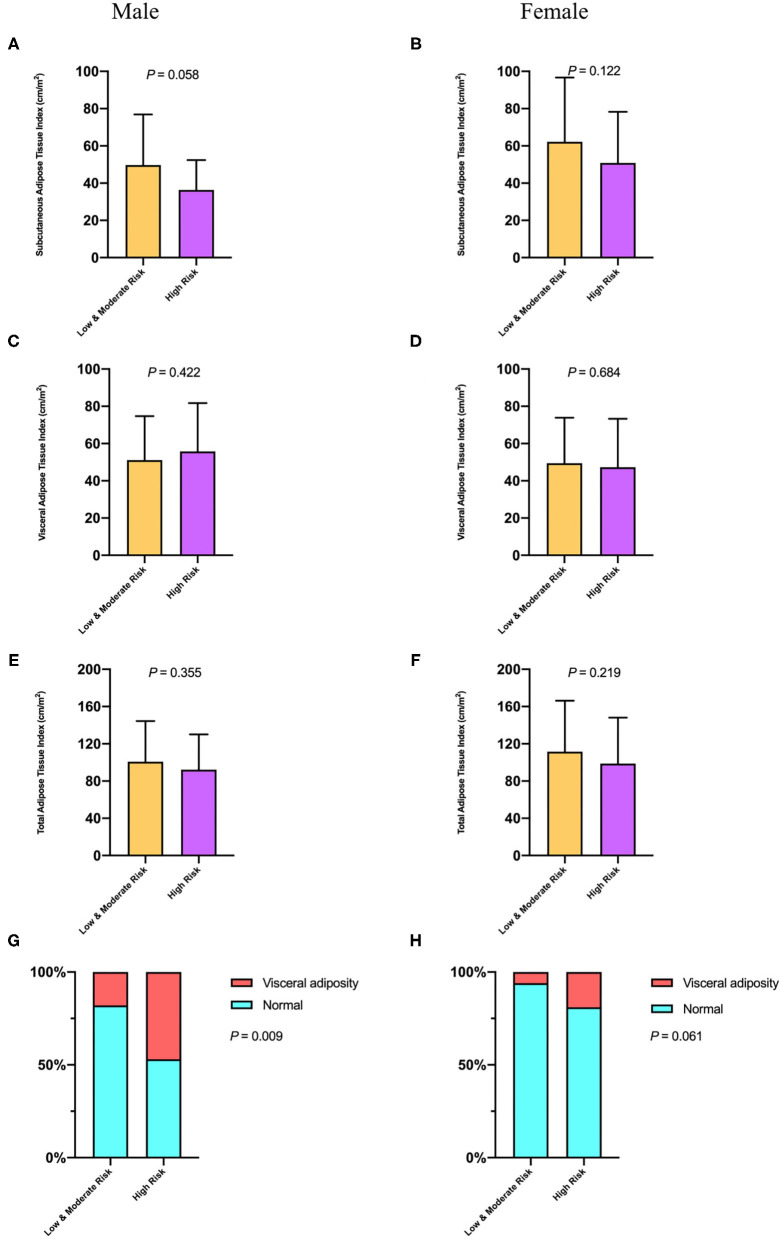
The comparison of subcutaneous adipose tissue index **(A,B)**, visceral adipose tissue index **(C,D)**, total adipose tissue index **(E,F)**, and visceral adiposity **(G,H)** in both genders with distinct malnutrition risk of hospitalized patients with cirrhosis.

It has been suggested that CT quantification unravels significant variations regarding adipose tissue distribution pattern by gender (19, 20). Specially, men store higher levels of VATI, whereas women have higher levels of SATI in the context of cirrhosis. Therefore, we further investigated the association between distinct adipose depots and RFH-NPT-based malnutrition risk by gender. As shown in Figure 2, there was no significant difference with respect to TATI (men: 100.80 ± 43.54 vs. 92.19 ± 37.94 cm^2^/m^2^, *p* = 0.355; women: 111.70 ± 54.65 vs. 98.19 ± 49.92 cm^2^/m^2^, *p* = 0.219), SATI (men: 49.50 ± 25.16 vs. 36.39 ± 15.96 cm^2^/m^2^, *p* = 0.058; women: 62.23 ± 34.50 vs. 50.91 ± 27.35 cm^2^/m^2^, *p* = 0.122), and VATI (men: 51.09 ± 23.60 vs. 55.80 ± 25.88 cm^2^/m^2^, *p* = 0.422; women: 49.42 ±24.48 vs. 47.28 ± 26.05 cm^2^/m^2^, *p* = 0.684) in both genders among patients with low or moderate and high risk of malnutrition. In contrast, the proportion of male patients embracing visceral adiposity was higher in high risk of malnutrition group compared with that in low or moderate group (47.27 vs. 17.86%, *P* = 0.009). Moreover, this disparity was of borderline statistical significance in women (19.05 vs. 5.88%, *P* = 0.061).

## Discussion

As far as we can determine, this is the first work to explore the association between CT-defined abnormal adiposity and validated tool (RFH-NPT) for screening malnutrition risk in hospitalized patients with cirrhosis. Our results implicated that high VSR that corresponds to visceral adiposity is associated with higher risk of malnutrition independent of BMI. Furthermore, the distribution of adipose tissue might alter more profoundly in comparison with other adiposity parameters when stratified by malnutrition risk.

Mounting evidence has proved that malnutrition serves as a predictor of morbidity and mortality in patients with cirrhosis (21). It is tempting to apply targeted interventions with the purpose of ameliorating malnourished status and relevant complications. Given no generalized modality currently exists regarding malnutrition risk screening, a cirrhosis-specific approach, referring to RFH-NPT, has recently been developed, used, and recommended by several hepatology centers and scientific societies (4, 22–25). Notably, among eight screening tools for detecting the risk of malnutrition in cirrhosis, RFH-NPT represents the most accurate with a high sensitivity of 97.4% and a fair specificity of 73.3% (26). In addition, our previous work showed that malnourished status assessed by RFH-NPT is closely associated with immune dysfunction (5). Taken together, we preferentially adopt RFH-NPT in this work for identifying high risk of malnutrition in our retrospective cohort.

In our work, the hospitalized patients with cirrhosis and high risk of malnutrition were at lower levels of BMI and prone to embrace higher proportion of visceral adiposity. Although BMI has been a concern in most researches evaluating clinical implications or outcomes in cirrhosis, it seems an inaccurate measurement of body composition. The main drawbacks of BMI include its inability to discriminate between muscle and adipose tissue as along with confounding impact of fluid retention in cirrhosis (8, 27). In a word, the fluid accumulation (e.g., large amount of ascites) might mask weight loss in patients with malnourished condition (6, 28). Consequently, it is recommended to perform a single crosssectional CT image or MRI, rather than BMI, as a non-invasive tool to estimate body composition. Moreover, it is feasible and available to most cirrhotics due to a routine request as screening for hepatocellular carcinoma by using crosssectional imaging.

Intriguingly, several studies have revealed the association between abnormalities in skeletal muscle and malnutrition risk in a wide array of pathological entities. Borges and colleagues showed that the presence of sarcopenia might predict comorbidities in 29%, and nutritional risk in 49% hospitalized patients with cancer (10). Akazawa et al. indicated that higher risk of malnutrition is associated with impaired muscle quality in terms of increased intramuscular adipose tissue of the quadriceps in elder inpatients (29). Notably, another work implicated that patients with cirrhosis and concomitant sarcopenia are predisposed to undernutrition (PG-SGA) and in need for nutritional care (11). Taken together, we and others have demonstrated that assessing body composition components, which includes both muscle and adipose tissue to stratify patients at high malnutrition risk and select more appropriate therapies.

In this work, we further confirmed that a higher VSR associates with high risk of malnutrition in patients with cirrhosis, whereas low subcutaneous adiposity or high visceral adiposity does not. Actually, our previous publication has already suggested that the distribution of adipose tissue, rather than the absolute value, represents a predominant risk factor of prognostication in cirrhosis (7). Marked differences between VAT and SAT have been observed regarding anatomic location, adipocyte size, insulin sensitivity, adipokines profile, and lipolytic capability (13). Adipocytes within VAT are responsible for secreting a variety of cytokines such as IL-1, TNF-α, and toxic-free fatty acids (FFAs) due to active lipolytic effect (30, 31). These FFAs are directly transported to the liver *via* portal vein, consequently resulting in oxidative stress, lipid peroxidation, and hepatocellular inflammation (32, 33). In contrast, SAT has been proved to uptake and deposit triglycerides, plasma FFAs, and responsible for producing leptin in charge of immune response and lipid metabolism (34–36). More recently, our results implicated that immune dysfunction measured by neutrophil-to-lymphocyte ratio (NLR) is associated with malnutrition risk estimated by RFH-NPT in cirrhosis (5). Furthermore, the expression of circulating IL-6 and IL-8 was positively correlated with increased NLR values (37). Taken together, we speculate that VSR might be more closely associated with chronic inflammation in patients with cirrhosis, which promotes the progression of malnutrition (38).

We acknowledge that there are limitations in this work. Firstly, we assured that cutoffs established in this work might not be generalized to other regions and populations. As a matter of fact, a myriad of cutoffs with respect to adipose tissue parameters have already been developed (19, 39). These dramatical disparities might be attributed to different ethnicities, analytic metrics, and also distinct pathologies. Secondly, we were unable to examine metabolic and nutritional profiles such as serum cytokines, nutrients, leptin, and insulin resistance because of the retrospective nature of this work. Thirdly, the sample size was relatively small in our work. Finally, we could not infer the causal relationship between visceral adiposity and the risk of malnutrition. Collectively, there is an urgent need to conduct randomized controlled trial to identify causality between these factors.

## Conclusions

In conclusion, the assessment of adipose tissue distribution by CT might potentiate the estimation of malnutrition risk in cirrhotics. It is pivotal to recognize visceral adiposity and develop targeted therapeutic strategies.

## Data Availability Statement

The raw data supporting the conclusions of this article will be made available by the authors, without undue reservation.

## Ethics Statement

The studies involving human participants were reviewed and approved by Ethics Committee of Tianjin Medical University General Hospital. The patients/participants provided their written informed consent to participate in this study.

## Author Contributions

XW, YL, MS, and CS equally contributed to the conception and design of the research. GG, WY, and YH contributed to the design of the research. ZY and CL contributed to the acquisition and analysis of the data. XF, BW, JZ, XZ, and KJ contributed to the interpretation of the data. CS drafted the manuscript. All authors critically revised the manuscript, agreed to be fully accountable for ensuring the integrity and accuracy of the work, and read and approved the final manuscript.

## Funding

This work was partly supported by the National Natural Science Foundation of China (Grant 81800531 to XZ) and by the Science and Technology Program of Tianjin (Grant 19ZXDBSY00020 to KJ).

## Conflict of Interest

The authors declare that the research was conducted in the absence of any commercial or financial relationships that could be construed as a potential conflict of interest.

## Publisher's Note

All claims expressed in this article are solely those of the authors and do not necessarily represent those of their affiliated organizations, or those of the publisher, the editors and the reviewers. Any product that may be evaluated in this article, or claim that may be made by its manufacturer, is not guaranteed or endorsed by the publisher.
