# Supplementary figures and images for: Visceral Adiposity Associates With Malnutrition Risk Determined by Royal Free Hospital-Nutritional Prioritizing Tool in Cirrhosis
Source: Front Nutr. 2021 Nov 24;8:766350. doi: 10.3389/fnut.2021.766350 (PMC8652121; doi:10.3389/fnut.2021.766350)

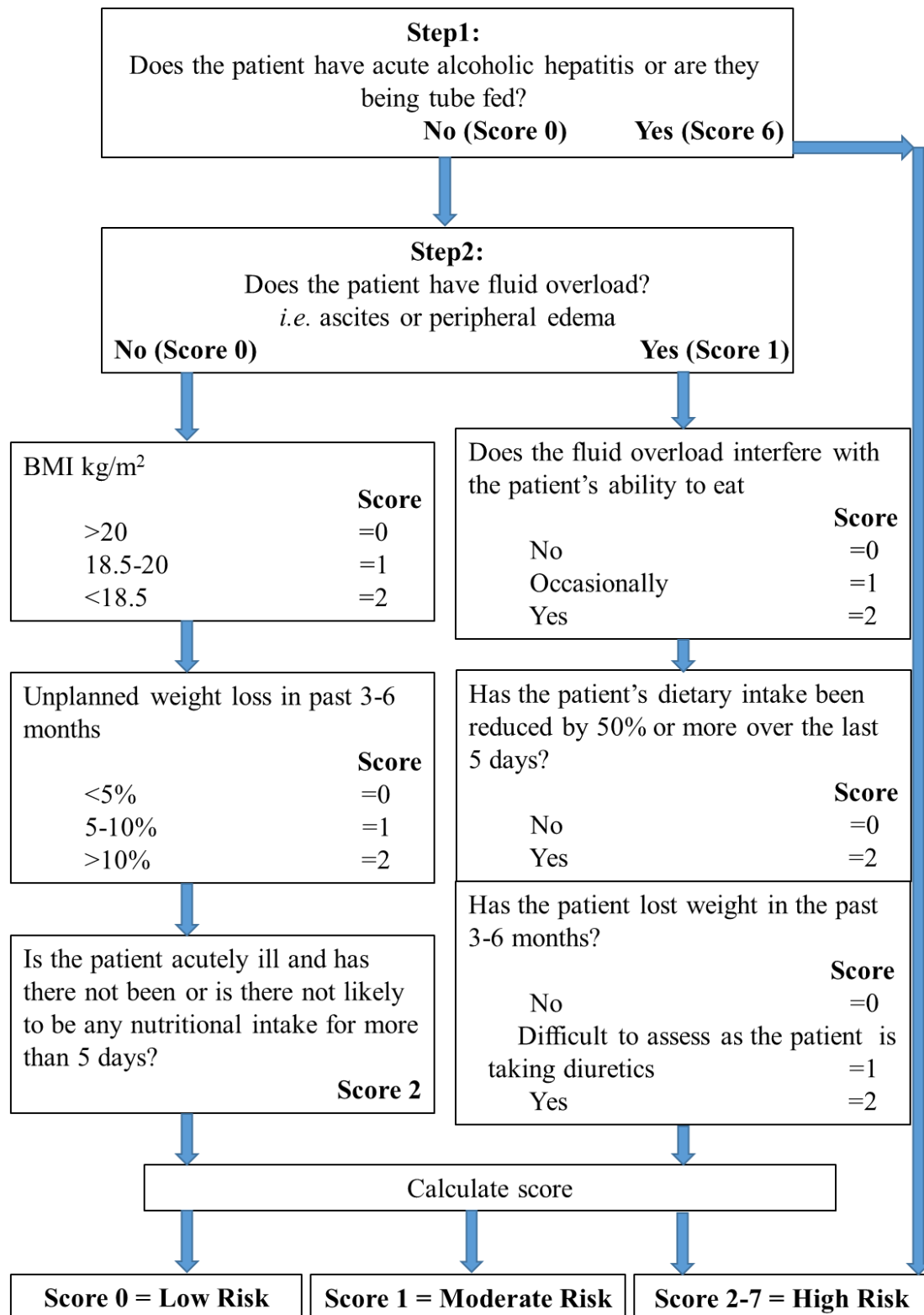

Supplement: Supplementary file 1 [file Image_1.pdf]
